# Antiphospholipid antibodies positivity as a potential risk factor for restenosis following arteriovenous fistula stenting in hemodialysis patients: a pilot study

**DOI:** 10.3389/fmed.2024.1497810

**Published:** 2025-01-03

**Authors:** Maxime Taghavi, Adrien Lengelé, Marc Laureys, Lucas Jacobs, Saleh Kaysi, Frédéric Collart, Anne Demulder, Joëlle Nortier

**Affiliations:** ^1^Department of Nephrology and Dialysis, Brugmann University Hospital, Université Libre de Bruxelles (ULB), Brussels, Belgium; ^2^Faculty of Medicine, Laboratory of Experimental Nephrology, Brussels, Belgium; ^3^Department of Radiology, Brugmann University Hospital, Brussels, Belgium; ^4^Laboratory of Hematology and Hemostasis, Brugmann University Hospital, Brussels, Belgium

**Keywords:** antiphospholipid antibodies, arteriovenous fistula, stenosis, stent, drug elusion coating, drug-eluted stent, intrastent restenosis, mTOR pathway

## Abstract

**Background:**

The arteriovenous fistula (AVF) is the preferred vascular access for hemodialysis. AVF stenosis is a common complication, often requiring balloon angioplasty. For recurrent stenosis, AVF stenting may be an option. Persistent antiphospholipid antibody (aPL) positivity is frequently observed in hemodialysis (HD) patients and is associated with AVF thrombosis and stenosis. This study aimed to evaluate AVF stent survival without stenosis in aPL-positive hemodialysis patients.

**Methods:**

A monocentric retrospective observational study was conducted on 35 patients who underwent AVF stenting between 1st January 2014 and 31st December 2023. The patients were divided into two groups: the aPL+ group [defined by a score of 3 or more based on the laboratory criteria of the 2023 ACR/EULAR for antiphospholipid syndrome (APS)] and the control group. Intrastent restenosis was defined as a chronic change in the AVFphysical examination or blood flow, confirmed by ultrasound (US) or angiography. Kaplan–Meier survival analysis was used to estimate the probability of stent survival without restenosis.

**Results:**

The prevalence of intrastent restenosis was significantly higher in the aPL+ group at 24 months. The Kaplan–Meier survival analysis showed a significantly lower probability of AVF stent survival without restenosis in the aPL+ group (age-adjusted Hazard Ratio, 2.13 [IC95%, 1.70–2.69]).

**Conclusion:**

To the best of our knowledge, we describe for the first time a statistically significant association between aPL+ and AVF intrastent restenosis. Intimal hyperplasia is a non-thrombotic lesion associated with aPL+ and is linked to the mammalian target of rapamycin (mTOR) signaling pathway. We hypothesize that aPL may contribute to intrastent restenosis by inducing intimal hyperplasia. Whether this phenomenon is mTOR-mediated and whether sirolimus-eluting stents or balloons could be a better option for aPL+ patients requires further study.

## 1 Introduction

According to the Kidney Disease Outcome Quality Initiative (KDOQI) Clinical Practice Guideline for Vascular Access, the creation of a native arteriovenous fistula (AVF) is considered the preferred method for establishing vascular access for adequate hemodialysis (HD) (1). AVF stenosis is a common complication typically located at the juxta-anastomotic region of the outflow vein. AVF stenosis may impact morbidity and mortality (2, 3). Ballon angioplasty is the first-line treatment option; however, stent placement can be used as a last-line treatment for recurrent AVF stenosis to maintain AVF patency (4).

Antiphospholipid syndrome (APS) is an autoimmune disease characterized by the persistent positivity of at least one antiphospholipid antibody (aPL), along with clinical manifestations defined in the 2023 ACR/EULAR classification criteria (5). APS has been associated with a variety of non-thrombotic manifestations such as vasculopathy, intimal hyperplasia, and intrastent restenosis after percutaneous coronary intervention (6, 7). The pathophysiology of stenotic lesions in APS involves endothelial dysfunction and activation of the mammalian target of rapamycin (mTOR) pathway (8, 9). Therapeutic inhibition of the mTOR pathway has been associated with better outcomes in APS-associated kidney involvement, both in native kidneys and after renal transplantation (9, 10). Given the role of the mTOR in vascular remodeling and intimal hyperplasia, its activation may similarly contribute to restenosis in AVFs.

The prevalence of persistent aPL positivity (with or without APS) is high in HD patients, ranging from 11 to 37% (11). The reasons for such a high prevalence are not well known, and persistent aPL positivity is sometimes considered an epiphenomenon in HD patients. Nevertheless, aPL positivity has been associated with AVF thrombosis in several studies (11–13) and inconsistently associated with AVF stenosis and maturation failure (11, 12).

While previous studies have demonstrated an association between antiphospholipid syndrome (APS) and in-stent restenosis in the coronary arteries, no data have been published regarding stent survival in AVFs among hemodialysis patients. Our study aimed to bridge this gap by investigating the potential link between persistent antiphospholipid antibody (aPL) positivity and in-stent restenosis in AVFs.

## 2 Materials and methods

### 2.1 Study design

This was a monocentric retrospective observational study. Institutional Review Board authorization was obtained from the Ethics Committee of Brugmann University Hospital – reference number CE2023/171, in accordance with the Declaration of Helsinki. The requirement for informed consent was waived by the Ethics Committee of Brugmann University Hospital because of the retrospective nature of the study.

We identified all AVF stenting procedures performed at our hospital between 1st January 1st 2014 and 31st December 31st 2023. Inclusion criteria were defined to ensure a homogeneous cohort and included patients over 18 years of age, undergoing hemodialysis through an AVF, who had received AVF stenting for recurrent stenosis, and had at least two available aPL assays. Exclusion criteria were carefully designed to minimize confounding factors and included the following: absence of available aPL assays or uninterpretable results, presence of thrombophilia other than antiphospholipid syndrome, active malignancy, aPL testing conducted under anticoagulation therapy (as it could interfere with accurate aPL measurements), acute thrombosis, and significant inflammatory states. These exclusion criteria aimed to eliminate factors that might have independently influenced the AVF outcomes or aPL measurements, ensuring the reliability of the analyses.

### 2.2 Stenting procedure

All AVF stenting procedures were performed by a single experienced interventional radiologist using a standardized technique. Prior to stenting, balloon angioplasty was systematically performed to address recurrent stenosis. Non-coated self-expanding metallic stents were used in all cases. Stent placement was guided by fluoroscopic imaging, ensuring precise positioning at the site of stenosis. There were no procedural variations between the aPL+ and control groups, ensuring homogeneity in the stenting approach.

### 2.3 Study groups

We classified the patients into two groups:

- The aPL+ group: defined by a score of 3 or more based on the laboratory criteria of the 2023 ACR/EULAR (meaning aPL persistent positivity at 12 weeks) (5).
- The control group: aPL-negative patients, defined by one or multiple negative aPL assays.

In our center, aPL assays were performed for all patients as part of a standard screening protocol at the start of chronic HD. If the first aPL assay was positive for any of the three aPL assays [lupus anticoagulant (LA), anti-β2 glycoprotein I antibodies (aβ2GPI), or anticardiolipin antibodies (aCL)], a 12-week confirmation assay was performed. The baseline characteristics were collected at the time of AVF stenting. The follow-up period was from AVF stenting until intrastent restenosis, loss of AVF, or loss to follow-up (death, transplantation, transfer to another center).

### 2.4 Endpoint

Intrastent restenosis was defined as a chronic change in the AVF physical examination or blood flow, confirmed by either ultrasound (US) or angiography. AVF stenosis was defined as a reduction of at least 50% in the vascular lumen diameter.

### 2.5 Statistical analyses

Normal distributed variables were presented as mean and standard deviation, and Student’s *t*-test was used for comparison. Variables with an asymmetric distribution were presented as the median and interquartile range (P25; P75), and the Mann–Whitney–Wilcoxon test was used for comparison. The significance level of the tests was set at 0.05. We employed Kaplan–Meier survival analysis to estimate the probability of stent survival without restenosis over time, accounting for censoring, and to compare survival curves between the groups. Due to the small sample size, non-parametric tests (e.g., Mann–Whitney U test) were used for the variables with asymmetric distributions, and Kaplan–Meier survival analysis accounted for the censored data in the survival outcomes. While no formal power calculation was performed, the observed significant associations suggested adequate power for the primary outcomes. Due to the limited sample size of our cohort, only the most clinically and statistically relevant variables were included in the multivariate analysis to avoid overfitting. Furthermore, the comparable prevalence of comorbidities such as diabetes and hypertension between the aPL+ and control groups minimized their impact as confounding variables. Statistical analyses were performed using SPSS software.

## 3 Results

**Figure 1:**
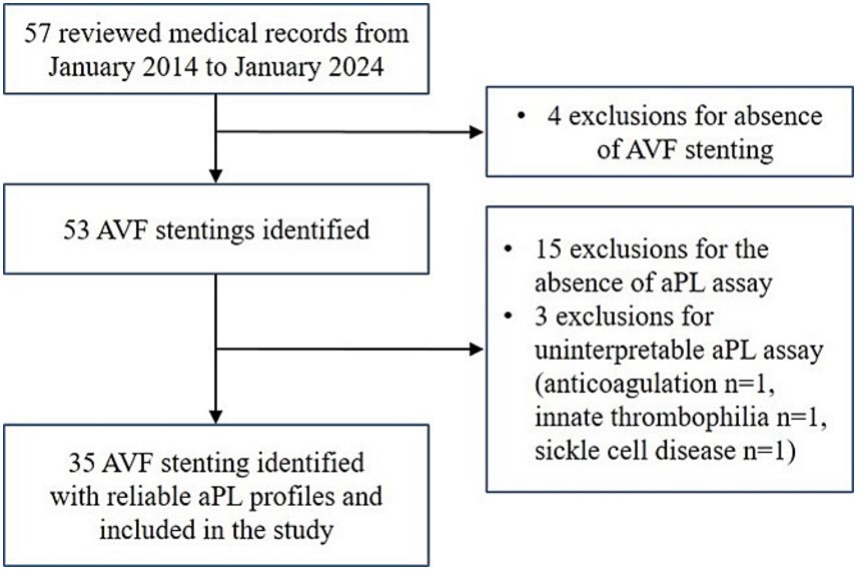
Study flow chart. AVF, arteriovenous fistula; aPL, antiphospholipid antibody.

From the 57 reviewed patient medical records, a total of 35 patients with AVF stenting met the inclusion criteria (the study flow chart is presented in Figure 1).

A total of eight patients were aPL+ and 27 patients were aPL–.Of the eight aPL+ patients, four were aPL carriers without APS and four were patients with APS. A total of six aPL+ patients had single lupus anticoagulant (LA) positivity, one patient had - single IgG aCL positivity, and one patient had double positivity (LA and high titers of IgG aCL). None of them were on oral or parenteral anticoagulants.

The aPL+ patients were older, with a median age of 68.8 years in the aPL+ group and 56.7 years in the control group (*p* = 0.028). The associated comorbidities were similar in both groups. Similarly, the AVF characteristics, stent characteristics, treatments, and laboratory findings were comparable in both groups (Table 1). All comorbidities were treated according to the guidelines in both groups.

**Table 1:** Baseline clinical, demographic, arteriovenous fistula characteristics, and stent characteristics of the patients included in the study.

	Total cohort (*n* = 35)	aPL+ (*n* = 8)	Controls (*n* = 27)	*p*-value
Demographics and comorbidities				
Age [year-old, median (P25; P75)]	59.8 (51.0; 68.3)	68.9 (61.4; 73.4)	56.3 (49.1; 64.2)	**0.028**
Sex male [%, mean (SD)]	77.1 (0.426)	88 (0.354)	74 (0.447)	0.442
BMI [kg/m^2^, mean (SD)]	25.0 (6.325)	24.12 (6.35)	25.3 (6.6)	0.307
Smoker [%, mean, (SD)]	51 (0.742)	50 (0.756)	52 (0.753)	0.952
Diabetes mellitus [%, mean (SD)]	49 (0.507)	50 (0.535)	48 (0.509)	0.929
Hypertension [%, mean (SD)]	94.3 (0.236)	88 (0.354)	96 (0.192)	0.361
HFrFE (LVEF <40%) [%, mean (SD)]	17.1 (0.382)	13 (0.354)	19 (0.396)	0.702
AVF and dialysis characteristics				
Distal AVF [%, mean (SD)]	54 (0.505)	63 (0.518)	52 (0.509)	0.608
AVF blood flow after stenting [mL/min, median (P25; P75)]	850.0 (437.0; 1162.5)	1,100 (812.5; 1762.5)	637.0 (420.0; 1,050)	0.213
HD vs. HDF [%, mean (SD)]	63 (0.490)	63 (0.518)	63 (0.492)	0.982
KT/V [mean (SD)]	1.52 (0.22)	1.45 (0.23)	1.54 (0.22)	0.329
Vintage [months, mean (SD)]	42.6 (35.7)	42.9 (39.2)	42.5 (35.4)	0.981
URR [%, median (P25; P75)]	73.8 (70.4; 78.4)	71.8 (67.8;80.6)	73.9 (70.8; 78.5)	0.582
Stent characteristics				
Stent Diameter [mm, mean (SD)]	8.2 (2.45)	8.14 (2.41)	8.22 (2.52)	0.945
Balloon diameter [mm, mean (SD)]	6.96 (2.45)	5.67 (3.27)	7.35 (2.11)	0.144
Length [mm, mean (SD)]	41.7 (14.1)	36.4 (0.2)	43.17 (15.1)	0.355
Restenosis prevalence at				
3 months [%, mean (SD)]	10 (0.305)	14 (0.378)	9 (0.288)	0.679
6 months [%, mean (SD)]	28 (0.455)	43 (0.507)	23 (0.429)	0.317
12 months [%, mean (SD)]	46 (0.508)	57 (0.535)	43 (0.507)	0.529
18 months [%, mean (SD)]	70 (0.465)	83 (0.408)	67 (0.483)	0.450
24 months [%, mean (SD)]	78 (0.424)	100 (0)	71 (0.463)	**0.010**
Treatments				
Statins [%, mean (SD)]	43 (0.502)	38 (0.518)	44 (0.506)	0.737
Anti-platelet therapy [%, mean (SD)]	69 (0.471)	63 (0.518)	70 (0.465)	0.684
Β-blocker [%, mean (SD)]	66 (0.482)	63 (0.518)	67 (0.480)	0.833
Laboratory findings				
Hemoglobin [g/dL, median (P25; P75)]	10.4 (9.4; 11.5)	11.2 (9.3; 12.1)	10.4 (9.4; 11.3)	0.432
Platelet count [×10^3^/μL, mean (SD)]	203.3 (42.8)	184.0 (131)	205.0 (71)	0.421
Mean Platelet Volume [fL, mean (SD)]	10.04 (1.28)	9.46 (1.14)	10.26 (1.29)	0.342
c-reactive protein [mg/L, mean (SD)]	6.3 (7.2)	4.8 (4.14)	6.7 (7.89)	0.522

aPL, antiphospholipid antibody; AVF, arteriovenous fistula; BMI, body mass index; HD, hemodialysis; HDF, hemodiafiltration; HFrEF, heart failure with reduced ejection fraction; LVEJ, left ventricle ejection fraction; SD, standard deviation, URR, urea reduction ratio. Bold text value are the statistically significant value.

The prevalence of intrastent restenosis was similar in both groups at 3, 6, 12, and 18 months. However, it was significantly higher in the aPL+ group at 24 months (Table 1). This association was also observed at 24 months when comparing the subgroups of the patients with APS to the controls (*p* = 0.01). When comparing the aPL asymptomatic carriers to controls, this association was even more pronounced, with a significantly higher rate of intrastent restenosis at 12, 18, and 24 months (*p* < 0.001, *p* = 0.005, and *p* = 0.01, respectively). Indeed, in this subgroup, all the patients experienced intrastent restenosis before 12 months.

**Figure 2:**
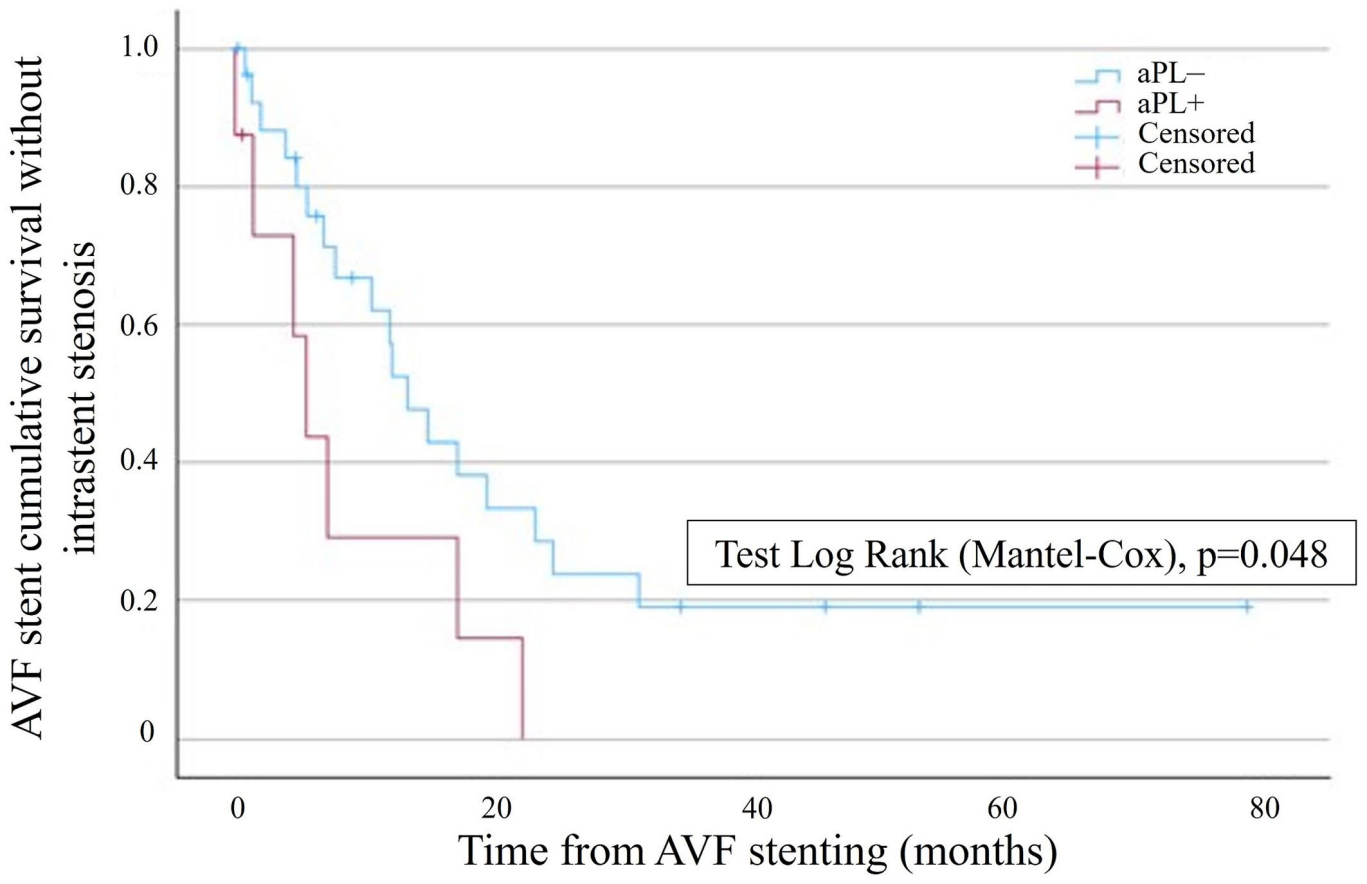
Kaplan–Meier survival analysis showing the probability of AVF stent survival without intrastent stenosis in the aPL+ group (red curve) and the control group (blue curve). AVF stent survival is on the ordinate, and time is on the abscissa.

The Kaplan–Meier survival analysis showed a significantly lower probability of AVF stent survival without restenosis in the aPL+ group (Figure 2). In the aPL+ group, only one patient did not experience intrastent restenosis (12.5%) and was censored after 0.6 months due to renal transplantation. In the control group, nine patients did not experience intrastent restenosis (33.3%), two patients completed the follow-up without experiencing intrastent restenosis, and seven patients were censored due to loss to follow-up. In this group, the mean time ± SD before the censoring was 17.1 ± 17.6 months. The Kaplan–Meier analysis accounted for the censored cases over time. By 12 months, one patient in the aPL+ group and seven patients in the control group were censored, primarily due to transplantation or loss to follow-up. These censored cases were minimal compared to the overall cohort size and, therefore, did not substantially limit the robustness of the survival estimates. However, it is important to note that the censoring at later time points might have still influenced the interpretation of the survival probabilities as fewer patients remained under observation.

**Figure 3:**
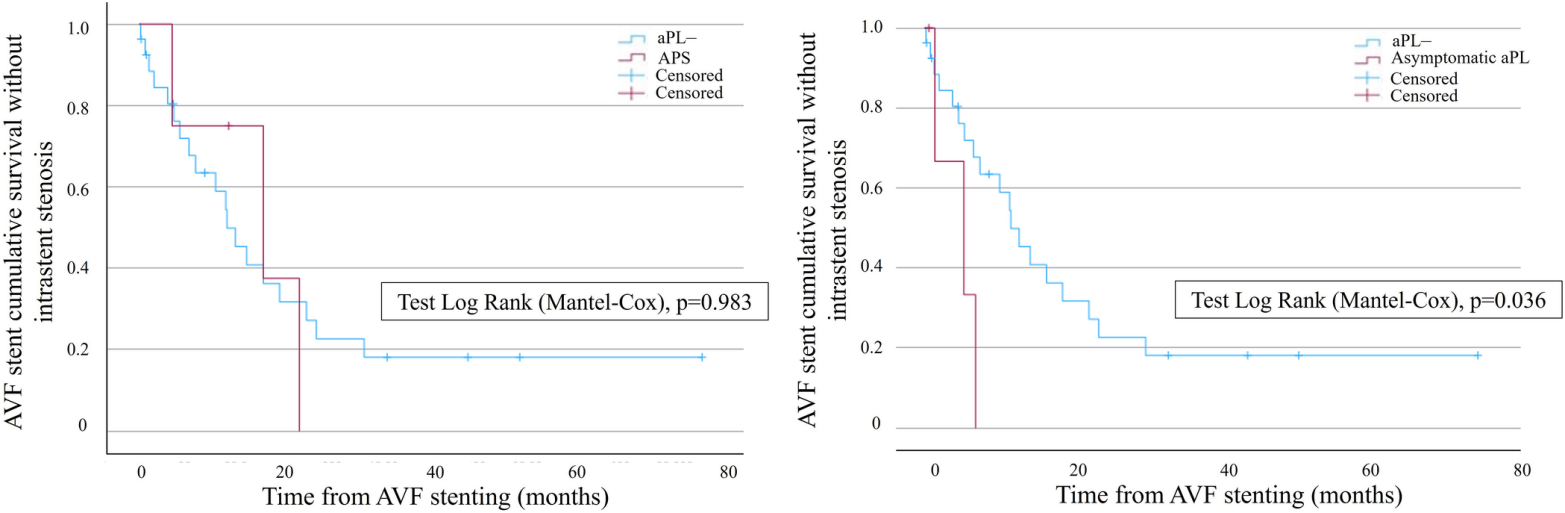
Kaplan–Meier survival analysis showing the probability of AVF stent survival without intrastent stenosis in the subgroups of the patients with APS (left curves) and aPL asymptomatic carriers (right curves). Blue curves represent the controls, whereas red curves represent the patients with APS (left graph) and aPL asymptomatic carriers (right graph), respectively. AVF stent survival is on the ordinate, and time is on the abscissa.

The mean time to the first event was 24.9 ± 6.78 months and 8.49 ± 3.12 months in the aPL– and the aPL+ groups, respectively (age-adjusted hazard ratio, 2.13 [IC95%, 1.70–2.69]). In the aPL+ group, the subgroup analysis of APS did not show a significant difference in terms of AVF stent survival without restenosis compared to the controls. However, the aPL asymptomatic carriers displayed a significantly lower probability of AVF stent survival without restenosis compared to the controls (Figure 3). The mean time to the first event was 23.7 ± 5.61 months and 4.72 ± 1.71 months in the control group and the aPL asymptomatic carrier group, respectively.

## 4 Discussion

Few studies have demonstrated that patients with APS are predisposed to high rates of intrastent restenosis in the coronary arteries after percutaneous coronary intervention, despite the similar use of drug-eluting stents in both APS and control groups (6).

This association has not been studied after the stenting of the AVF vasculature. Since AVFs have different diameters, blood flow, and vascular structures compared to the coronary arteries, we investigated this association in our HD population in cases where AVF stenting was used to treat recurrent stenosis. To the best of our knowledge, we describe for the first time a statistically significant association between persistent aPL positivity and AVF intrastent restenosis. In our cohort, stent survival without restenosis was lower in the aPL+ group compared to the control group, with the mean time to the first event being almost three times shorter in the aPL+ group (age-adjusted hazard ratio, 2.13 [IC95%, 1.70–2.69]). In addition, the 24-month restenosis rate was significantly higher in the aPL+ group, indicating that this complication can occur long after the stenting procedure.

The pathophysiology of intrastent restenosis is complex and not fully understood. It involves inflammation in response to endothelial injury, smooth muscle cell migration and proliferation, extracellular matrix formation, endothelial dysfunction, and the occurrence of intimal hyperplasia (4, 13, 14). Drug-eluting balloons (DEB) and drug-eluting stents (DES) (primarily paclitaxel-coated) have been used to improve post-procedure AVF patency and reduce the reintervention rate compared to conventional angioplasty in HD patients (15–17). mTOR inhibitor-coated balloons or stents, commonly used in percutaneous coronary intervention, have not been studied in the AVF vasculature. The IMPRESSION Study is a prospective, multicenter, double-blind, randomized controlled clinical trial designed to assess the effectiveness of sirolimus-coated balloons compared to conventional balloon angioplasty in improving the patency of AVFs after angioplasty (18).

Interestingly, intimal hyperplasia is a well-known non-thrombotic histological lesion associated with APS, particularly in APS nephropathy (7). This process involves endothelial dysfunction and the activation of the mTOR signaling pathway, which plays a central role in vascular remodeling and intimal hyperplasia—key processes in restenosis (9). In the context of aPL-associated coronary artery disease, stents coated with mTOR inhibitors have been suggested as a potential treatment for patients with APS and myocardial infarction (19). Given the association between antiphospholipid antibodies, endothelial dysfunction, and intimal hyperplasia, the use of sirolimus-eluting stents or balloons targeting the mTOR pathway may offer a promising strategy for aPL+ patients. This approach has shown efficacy in coronary interventions and could be extended to vascular complications in HD populations. We hypothesize that antiphospholipid antibodies may be associated with intrastent restenosis in HD patients by inducing endothelial dysfunction and intimal hyperplasia. Future randomized trials are essential to optimize treatment strategies and stent selection for aPL+ patients.

In addition, another pathophysiological mechanism of intrastent restenosis could be the occurrence of accelerated atherosclerosis in vessel areas not covered by stents. Indeed, aPL has been associated with accelerated atherosclerosis, as well as cardiovascular disease and peripheral artery disease (20). Thus, an atherogenic hypothesis has been proposed by some authors, which may help explain the link between aPL and fistula occlusion. This could contribute to vascular dysfunction beyond the stented segment in AVFs, potentially narrowing other vessel segments and impacting patency. Addressing systemic vascular health alongside restenosis, through interventions such as statins or anti-inflammatory treatments, could improve outcomes in aPL-positive patients. Further research is needed to explore this interplay and optimize management strategies. In our cohort, we did not find any difference between the groups in terms of cardiovascular disease or related treatments.

Our findings have potential clinical implications for the management of HD patients undergoing AVF stenting. Firstly, routine screening for aPL positivity in HD patients could help identify individuals at higher risk for intrastent restenosis. In aPL-positive patients, closer monitoring of AVF functionality, including frequent ultrasound assessments, might be warranted to detect early signs of restenosis. Secondly, the exploration of alternative endovascular approaches, such as the use of sirolimus-eluting stents or balloons, could represent a promising therapeutic strategy for aPL-positive patients, as these devices target mTOR activation, a potential mechanism involved in restenosis. Lastly, integrating aPL status into the clinical decision-making process could help stratify risk and personalize care strategies, optimizing outcomes for this high-risk population. Future prospective studies are needed to validate these approaches and further define the role of mTOR inhibitors in aPL-positive patients.

The key strength of our study is the rigorous inclusion criteria as we included only patients with persistent aPL positivity (12-week confirmation assay). In addition, the groups were homogenous in terms of the AVFs and stenting characteristics. Moreover, all the stents were placed by the same interventional radiologist and were non-coated, limiting heterogeneity. While the baseline characteristics, including diabetes and hypertension, were largely similar between the aPL+ and control groups, these comorbidities are known to influence vascular outcomes and may affect the risk of AVF restenosis. Their comparable prevalence between the groups minimized potential confounding, and all comorbidities were managed according to the standard guidelines, reducing variability in the treatment approaches. Future studies should incorporate multivariate analyses to further adjust for these variables and confirm our findings. However, this study has several limitations because of its retrospective monocentric nature and the limited number of patients. The small cohort size led to bias, with a significant age difference between the aPL+ and control groups (*p* = 0.028). Although other demographic characteristics and comorbidities were similar, age might have influenced the risk of intrastent restenosis. In our analysis, age was accounted for through an adjusted hazard ratio (age-adjusted HR: 2.13 [95% CI, 1.70–2.69]). However, due to the limited sample size, we could not fully exclude the potential influence of age on our results. This represents a limitation of our study that warrants further investigation in larger cohorts. In addition, the short follow-up period for some patients might have limited our ability to fully assess long-term restenosis outcomes. Future studies should focus on prospective, multicenter cohorts with larger sample sizes to validate these results. Furthermore, the small sample size reduced statistical power and might have overestimated the effect sizes, particularly in the subgroup analyses. Although the results are promising, it is difficult to extend them to the general HD population. These results must be confirmed in a larger cohort and multicentric study. Nevertheless, the prospects are promising. Future studies could prospectively evaluate mTOR-coated stents or balloons and aPL assays in HD patients.

## 5 Conclusion

We report for the first time a statistically significant association between persistent aPL positivity and AVF intrastent restenosis. This association was stronger at 24 months. Detection of aPL positivity could be a useful tool for clinicians in assessing the risk of AVF intrastent restenosis. Further studies are warranted to confirm this association. In addition, because APS is associated with mTOR activation, further studies should focus on evaluating the role of the mTOR pathway in aPL-associated intrastent restenosis in HD patients and on assessing mTOR inhibitor-eluting balloons or stents.

## Data Availability

The raw data supporting the conclusions of this article will be made available by the authors, without undue reservation.
